# Careful with That Axe, Gene, Genome Perturbation after a PEG-Mediated Protoplast Transformation in *Fusarium verticillioides*

**DOI:** 10.3390/toxins9060183

**Published:** 2017-05-31

**Authors:** Valeria Scala, Alessandro Grottoli, Riccardo Aiese Cigliano, Irantzu Anzar, Marzia Beccaccioli, Corrado Fanelli, Chiara Dall’Asta, Paola Battilani, Massimo Reverberi, Walter Sanseverino

**Affiliations:** 1 Consiglio per la Ricerca in Agricoltura e l'Analisi dell'Economia Agraria - Research Center for Plant Protection and Certification, 00156 Roma, Italy; valeria.scala@crea.gov.it; 2 Department Environmental Biology, Università Sapienza, 00185 Roma, Italy; alessandro.grottoli@uniroma1.it (A.G.); marzia.beccaccioli@uniroma1.it (M.B.); corrado.fanelli@uniroma1.it (C.F.); 3 Sequentia-Biotech SL, 08028 Barcelona, Spain; raiesecigliano@sequentiabiotech.com (R.A.C.); ianzar@sequentiabiotech.com (I.A.); wsanseverino@sequentiabiotech.com (W.S.); 4 Department Food Chemistry, Università di Parma, 43120 Parma, Italy; chiara.dallasta@unipr.it; 5 Department Sustainable Crop Production, Università Cattolica del Sacro Cuore, 29100 Piacenza, Italy; paola.battilani@unicatt.it

**Keywords:** gene deletion, parasexual cycle, in vitro evolution, mitotic recombination, bioinformatics, *Fusarium* database

## Abstract

*Fusarium verticillioides* causes ear rot disease in maize and its contamination with fumonisins, mycotoxins harmful for humans and livestock. Lipids, and their oxidized forms, may drive the fate of this disease. In a previous study, we have explored the role of oxylipins in this interaction by deleting by standard transformation procedures a linoleate diol synthase-coding gene, *lds1*, in *F. verticillioides*. A profound phenotypic diversity in the mutants generated has prompted us to investigate more deeply the whole genome of two *lds1*-deleted strains. Bioinformatics analyses pinpoint significant differences in the genome sequences emerged between the wild type and the *lds1*-mutants further than those trivially attributable to the deletion of the *lds1* locus, such as single nucleotide polymorphisms, small deletion/insertion polymorphisms and structural variations. Results suggest that the effect of a (theoretically) punctual transformation event might have enhanced the natural mechanisms of genomic variability and that transformation practices, commonly used in the reverse genetics of fungi, may potentially be responsible for unexpected, stochastic and henceforth off-target rearrangements throughout the genome.

## 1. Introduction

Species belonging to the genus *Fusarium* can grow successfully on a variety of substrates, tolerate diverse environmental conditions and have high levels of intraspecific genetic and genotypic diversity [1]. Notably, the genomes of fungi favor the evolution of features that increase plasticity and can enable rapid adaptation to changing environmental conditions [2,3]. In general, sexual reproduction ensures variability in organisms; when individuals are produced asexually, the frequency and degree of variability among the progeny is greatly reduced. In several fungi, the parasexual processes “can and do produce variants by means of mutations in the absence of any sexual process” [4]. The prerequisite to parasexual gene recombination is the establishment of heterokaryon; this condition can occur for mutation, in multinucleated cells, anastomosis of hyphae that are genetically different and subsequent migration of nuclei [5]. As described in *Aspergillus nidulans* (Winter, 1884 in [6]), the standard parasexual cycle starts with the formation of a heterokaryon (plasmogamy) followed by the fusion of the two haploid genomes (karyogamy). The diploid nuclei may be unstable and consequently produce haploid or aneuploid segregants through mitotic recombination and chromosomal nondisjunction [7,8]. Sexual and parasexual recombination allow fungi to exist in heterokaryotic, homokaryotic, recombinant haploid or diploid nuclei conditions. If these nuclear states do occur in vegetative hyphae and conidia, they may be important drivers of genetic variation [5] and may influence the phenotypic and genotypic characteristics of the single strain.

Among the genus *Fusarium*, *F. verticillioides* (Sacc.) Nirenberg (teleomorph *Gibberella moniliformis* Wineland) is the causal agent of the ear and stalk rot of maize. *F. verticillioides* belongs to the ‘‘African” clade of the *Fusarium fujikuroi* species complex [9]. *F. verticillioides* is frequently isolated from maize grown in Italy [10]. Tissue invasion is often asymptomatic even in the presence of massive growth inside the kernels [11]. *F. verticillioides* may produce fumonisins during host invasion. Fumonisins are classified into more than 90 analogues A-, B-, C- and P-series [12], with the sole FB1 reputed by the International Agency for the Research on Cancer as a class 2B carcinogen [13] because of its toxic effects in animals and as a possibly carcinogenic in humans [14,15]. 

In a previous work [16], we established several physiological-related roles for the oxylipins produced by the enzyme linoleate diol synthase 1 (LDS1) in *F. verticillioides*. Notably, LDS1-oxylipins resulted in being negative regulators of growth, conidiogenesis, aggressiveness and fumonisin synthesis in *F. verticillioides*. These results were inferred by studying the “average” phenotype and physiology of the *lds1-*deleted mutants. Indeed, multiple phenotypes emerged. Some of these features, for instance the lack or strong limitation in the synthesis of specific oxylipins (i.e., 8,13-diHODE), were closely linked with *lds1* gene deletion, while some others were reasonably not. 

Why may transformed fungi give rise to genotypic and phenotypical variations further than those trivially derived by the deletion of the gene target? To answer this question, we considered that in fungi and in other phyla [17,18,19], in vitro propagation determines the accumulation of mutations within the genome. Genetic variations can be an indispensable tool as an evolutionary response to in vitro selection [20]. Moreover, deletions or rearrangements, associated with transgene insertion, further increase the likelihood of alterations to the phenotype [21]. Actually, gene deletion is a molecular approach effective in identifying genes or gene clusters’ function in filamentous fungi inter alia. Genes can be knocked-out by homologous recombination with a deletion cassette in which the 5′ and 3′ UTR of the target gene were placed alongside a gene coding for a selectable marker (e.g., resistance to an antimycotic). These cassettes can be inserted into fungal protoplasts by PEG-mediated transformation [22].

The object of our study was establishing, by a bioinformatics approach coupled to standard biomolecular techniques, the rate of genome alteration of *F. verticillioides* after the event of PEG-mediated transformation of protoplasts with a gene deletion cassette. To pinpoint the genetic variations caused by the PEG-mediated transformation, we considered the basal rate of variation induced by the sub-culturing procedures and the potential effects of the parasexual cycle in *F. verticillioides*.

## 2. Results

### 2.1. Phenotypic Variations Within lds1-Deleted Strains

**Figure 1:**
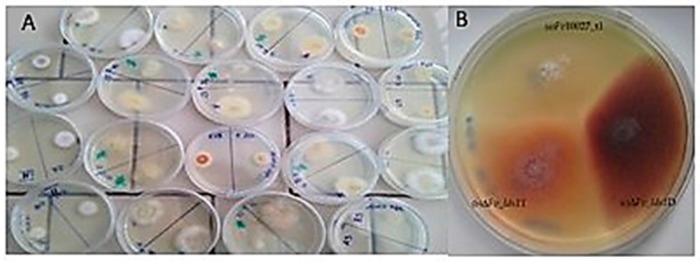
(**A**) Set of 20 mutants originated by PEG-mediated transformation of protoplasts of Fv10027. (**B**) Colony morphology of ΔFv_*lds1*D, ΔFv_*lds1*D T and Fv10027 strains.

Scala et al. [16] demonstrated that *lds1* deletion severely affects fungal morphogenesis, virulence and secondary metabolism in *F. verticillioides*. Indeed, the deletion of a copy of the *lds1* gene affected fungal phenotype well beyond the effects directly ascribable to the role of this gene. A set of mutants (n. 20) produced after the *lds1* gene deletion showed different phenotypes when grown on solid media, ranging from pink hyphae submerged into agar medium to white fluffy aerial hyphae (Figure 1A,B). The mutants also differ in the ability to degrade the cellophane disk (data not shown). Most important, *lds1*-deleted strains (a subset of four mutants, D, T, M, L, has been more deeply investigated) showed intra-individual differences in conidiation ability, conidia germination, growth in solid media, oxylipins and fumonisins production under in vitro conditions (Table 1). Further investigations, under in vivo conditions have been performed on two *lds1-*deleted strains: (ΔFv_*lds1*D and ΔFv_*lds1*T), which presented single insertion of the *hph* cassette (Table 1, Supplementary Figure S1). Some parameters such as oxylipin gene expression, fungal growth and fumonisins production were also verified in the control mutant (strain transformed with the hygromycin B resistance cassette alone; FvHph^+^) (Table 1), resulting in significant variations (student *t*-test; *p* < 0.01) in amount in comparison with the WT strain. 

**Figure 2:**
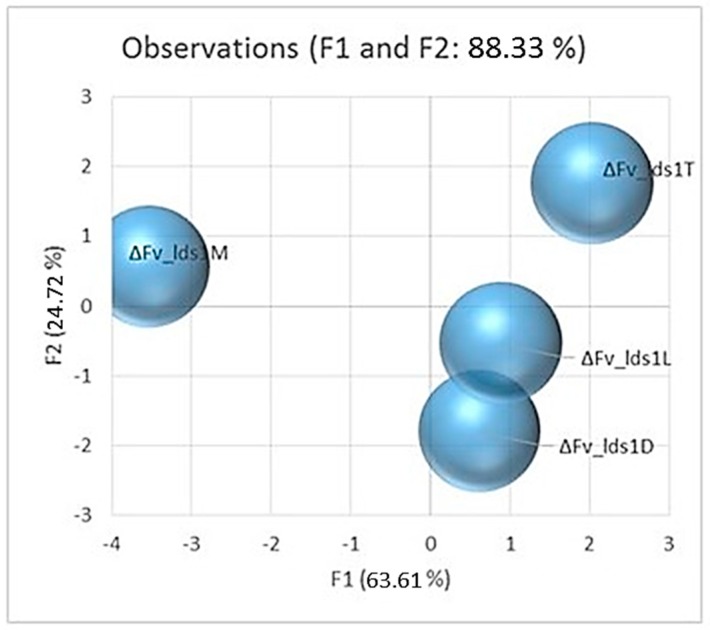
PCA score plot of data generated by the analysis of several physiological parameters such as fungal growth, conidiogenesis, fumonisins production and antioxidant enzymes activities inter alia (see the description of Table 1). Observations were clustered according to the strain (ΔFv_*lds1*D, L, M and T).

Some physiological parameters checked are directly linked to *lds1*-deletion (i.e., oxylipins); others can be reputed under the hormonal control of oxylipins (e.g., conidiogenesis), whilst others are not trivially controlled by these molecules (e.g., antioxidant enzymes). Indeed, these tables pinpointed clear and significant differences with the WT strain and, most interestingly, between the mutant strains (Table 1). PCA analysis performed on the whole dataset (Figure 2) confirmed the distinctive features among the four mutants, further validating the specificity of the parameters evaluated. A picture emerged that indicated profound modifications in the phenotype, i.e., in the physiology and in the morphology of the colonies, caused by the PEG-mediated transformation of Fv10027 protoplasts with the *lds1*-deletion cassette.

### 2.2. Variant Calling and Annotation

The evaluation of genome stability, caused by PEG-mediated transformation, was performed by a comparative whole genome re-sequencing analysis of the wild type strain (Fv10027), as well as of two *lds1*-deleted mutants (ΔFv_*lds1*T and D) and the control mutant FvHph^+^. Thus, we re-sequenced the Fv10027 (t0 and t1; check the Methods for the description), ΔFv_*lds1*T and ΔFv_*lds1*D genomes (NCBI SRA Project Accession SRP076367) and FvHph^+^ genome for characterizing the genetic diversity within our strains. The main goal was the identification of genetic variations compared to the reference *F. verticillioides* genome (FV7600), including single nucleotide polymorphisms (SNP), small insertions and deletions (deletion/insertion polymorphisms (DIPs)) and structural variants (SV). Variant calling analysis was performed using resequencing data of four different strains and, as reference genome, the official reference Fv7600 [23]. 

**Figure 3:**
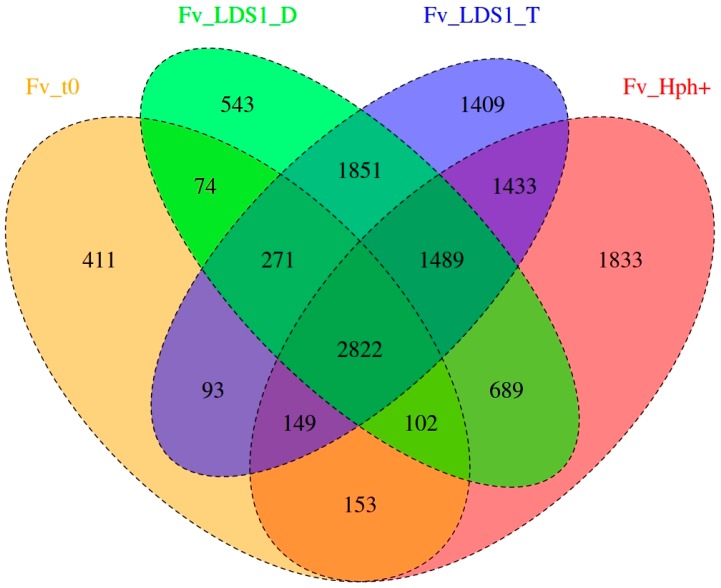
Venn diagram showing the number of variants present, common and exclusive to the four samples (Fv10027_t0, FvHph^+^, ΔFv_*lds1*D and ΔFv_*lds1*T) in comparison with Fv10027_t1.

**Figure 4:**
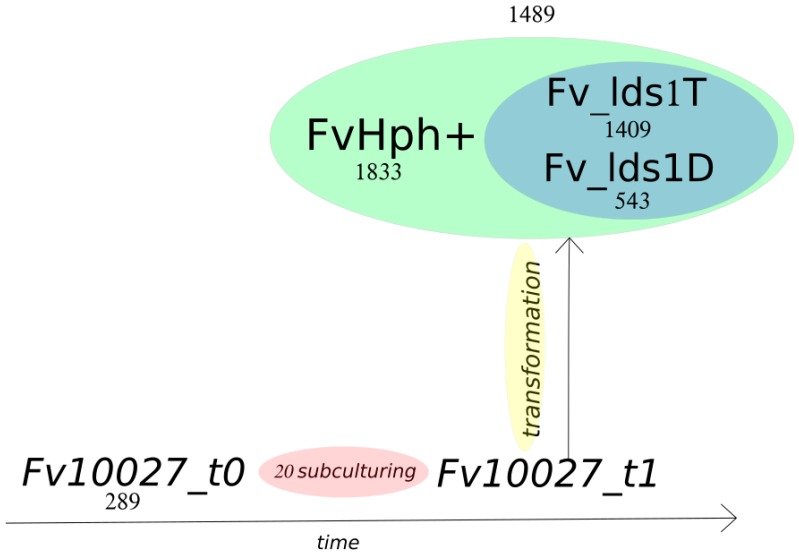
Temporal distribution of strains and variation numbers. Fv10027_t0 represents the strain derived from the original culture obtained from the fungal germplasm bank of ISPA-CNR (Bari, Italy); Fv10027_t1 is originated from Fv10027_t1 after different monoconidial subcultures (#20). Fv10027_t1 was used to generate ΔFv_*lds1* and FvHph^+^ mutant strains. The amount of variations with respect to Fv10027_t1 (values below each strain) and the numbers of common variants between ΔFv_lds1 mutant strains and FvHph^+^ (value above the mutant strains) are shown.

Notably, two WT (Fv10027_t0; Fv10027_t1), two lds1-deleted mutants (ΔFv_*lds1*T and ΔFv_*lds1*D) and the control mutant (FvHph^+^) strains were used. Fv10027_t0 represented our “starting culture”, i.e., spores deriving from monoconidial culture obtained from a germplasm collection (see the Materials section), whereas Fv10027_t1 represented the mycelia obtained after twenty subculturing passages from Fv10027_t0 (see the Methods for the description). Fv10027_t1 conidia were used for obtaining protoplasts and deleting the *lds1* gene [16]. For this reason, the genome of Fv10027_t1 has been used as a reference (see the chapter on genome reconstruction below) for the variant calling analysis. This analysis highlighted genetic variations of Fv10027_t1 with the other strains (Fv10027_t0, ΔFv_*lds1*T, ΔFv_*lds1*D and FvHph^+^). The open source pipeline SUPER-W [24] has been used for this purpose. Overall, 285,588 raw variants have been found: namely, 273,744 are SNPs, and 11,844 are DIPs. Then, a variant quality filtering was performed to filter out low quality variants and all common variants between Fv10027_t0, Fv10027_t1, ΔFv_*lds1*T, ΔFv_*lds1*D and FvHph^+^ (i.e., 251,355 SNPs and 11,614 DIPs). Through this tool, a PHRED quality of ≥30 and a depth filter of ≥5 were used to filter out false positives, as shown in Table 2. From this filtering process, 18,964 variants emerged: 18,802 SNPs and 162 DIPs (Table 2). All of the SNPs and DIPs (private and common) coming from Fv10027_t1 (reference strain) were also removed for pinpointing informative variants coming exclusively from the transformation process. Through this, we obtained 13,202 variants ascribable to the transformation. To further stress the difference existing among our transformed versus naive strains, a Venn diagram consisting of separating those variants according to their presence or absence in one or more samples (Figure 3) and a timeline of the transformation process were shown (Figure 4). All of the variants, generally heterozygotes, were also annotated for their possible effects. Of 13,202 variants, 13,086 are SNPs, and 116 are DIPs (49 insertions and 67 deletions). Dividing it for their effect, 617 are missense, 581 are silent, 21 are nonsense (stop codon gained), while 9 have a frameshift effect (Table 3). Furthermore, 119,426 raw SV were found. A specific SV quality filtering was performed to filter out common and low quality SV and a depth filter of ≥5. From the filtering step, 8329 SV emerged, and a 535-bp SV has been shown even by standard procedures (Supplementary Figure S2).

### 2.3. Significance of Variations in ΔFv_lds1 Mutant Strains

**Figure 5:**
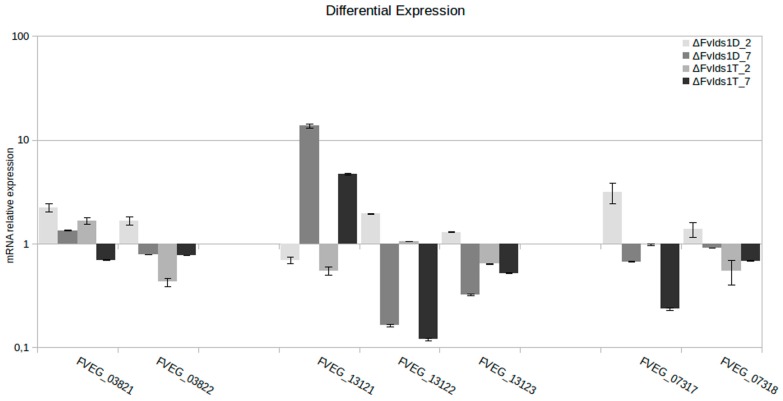
mRNA relative expression of different genes hypothetically affected by variations in mutants ΔFv_*lds1*D and ΔFv_*lds1*T compared to Fv10027_t1. Specifically, FVEG_03821 and FVEG_03822 were affected by DIP1; FVEG_16695 was affected by SNP5; FVEG_13121, FVEG_13122 and FVEG_13123 were affected by DIP4; FVEG_07317 and FVEG_07318 were affected by Structural Variant 1 (SV-1). The bar represents the mean value ± SE of nine repetitions (3 biological × 3 technical).

Since we aimed at evaluating the genome perturbation triggered by the sole transformation procedures, for subsequent analysis, we focus on the sole haplotypic variations not attributable to subculturing. These (#9248) represented the set of the private and common variants in ΔFv_*lds1*T, ΔFv_*lds1*D and FvHph^+^, namely, 9169 SNPs and 79 DIPs (48 deletions and 31 insertions). High effect sets of variations, 15 stop gained and nine frameshifts, were described affecting several genes as shown in Table 3. The presence of several variations was cross-validated by standard procedures, e.g., Sanger sequencing and end-point PCR analysis (Supplementary Figure S2). All primers were designed using the reconstructed genome Fv10027_t1 (Supplementary Table S3). The bioinformatics analysis reported that variations found in ΔFv_*lds1*D and ΔFv_*lds1*T (respect to Fv10027_t1) could affect the expression of different genes present within the specified genome position (Table 3). RT-qPCR was used to validate the bioinformatics results. Among the genes affected by genomic variations, we analyzed the relative expression of FVEG_03821, FVEG_03822, FVEG_13121, FVEG_13122, FVEG_13123, FVEG_07317 and FVEG_07318 (Figure 5). Results indicated a profound alteration of gene expression in ΔFv_*lds1*D and ΔFv_*lds1*T strains at two and seven days after inoculation (dai) compared to the WT strain. DIP1 (DIP-variation) produced a differential expression in both affected genes FVEG_03821 and FVEG_03822 (respectively, *p* < 0.05 and *p* < 0.001). Specifically, in ΔFv_*lds1*D strain, FVEG_03821 and FVEG_03822 expression is higher with respect to Fv10027_t1 (*p*-value < 0.05). In the ΔFv_*lds1*T strain, FVEG_03821 is more expressed, whereas FVEG_03822 is less expressed with respect to the WT (Figure 5). In ΔFv_*lds1*D, DIP4 negatively affected the expression of FVEG_13121 with respect to the WT, whereas FVEG_13122 and FVEG_13123 are more expressed with respect to the WT. In ΔFv_*lds1*T, FVEG_13121 is down-modulated in comparison to the WT strain; the expression of the FVEG_13122 is similar to the WT; and FVEG_13123 is less expressed with respect to the WT (Figure 5). The SV1 variation, a 534-bp deletion, causes the loss of the stop codon in FVEG_07317 and the loss of the 5′ upstream of FVEG_07318. This SV variation affected in ΔFv_*lds1*D the expression of FVEG_07317 and FVEG_07318 genes that are more expressed with respect to WT. In ΔFv_*lds1*T, the FVEG_07317 and FVEG_07318 genes are less expressed with respect to the WT (*p* < 0.01) (Figure 5).

### 2.4. Multiple Nuclei as a Natural Source of Genome Variation

**Figure 6:**
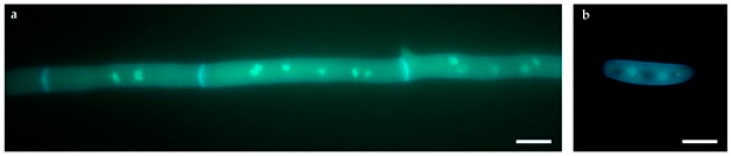
Fluorescence microscopy images of *Fusarium verticillioides* 10027 (**a**) hyphae and (**b**) asexual spore stained with DAPI and calcofluor (bar = 10 μm).

Although not obvious, the presence of multiple nuclei within individual hyphal compartments or within asexual spores is usual in the fungal kingdom and notably in the Ascomycota division [25]. Indeed, the single hyphal compartment (Figure 6a), as well as conidia (Figure 6b) of Fv10027 and of the reference strain Fv7600 presented multiple nuclei (2.83 ± 0.3 and 2.38 ± 0.1, respectively). Most intriguingly, SUPER-W [24] allowed identifying multiple haplotypes in the variants found (improperly called previously as “heterozygotes”) as shown in Table 4. We identified a background rate of heterozygous variants that is present in the WT strain (Fv10027_t0). This could indicate that multiple haplotypes occurred naturally in this species and that this phenomenon can be augmented by subculturing procedures. This may occur for the simultaneous presence of multiple nuclei per single compartment as shown in Figure 6a,b. In relation to this, the presence of multiple haplotypes in the variants is suggestive of the occurrence of the parasexual cycle (creating nuclei with different levels of ploidy) even in our WT strain.

### 2.5. Genome Reconstruction: A New Frontier for Downstream Analysis

**Figure 7:**
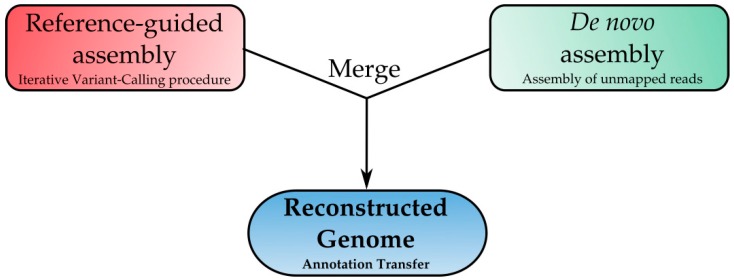
Step 1, reference guided assembly: through iterative SUPER-W software read mapping and variant calling processes, a new reference genome is generated with the detected and accurately-filtered variants. By an iterative process, we mean that it is a recursive process; the read mapping and variant calling is completed six times. Step 2, *de novo* assembly: through a *de novo* assembly strategy, *de novo* contigs are assembled into “raw” contigs and then filtered with a BLAST search generating filtered contigs. Then, the reconstructed genome (with the detected variants included) from Step 1 and the de novo contigs assembled at Step 2 are merged using paired-end and split-read information, having as the result the final reconstructed genome. Step 4, annotation transfer: the official annotations are transferred into the reconstructed genome.

The emergence of significant differences between our wild type Italian strain (Fv10027) and the American strain (Fv7600) and the planned downstream analyses (see Methods’ Molecular Analysis section) pointed out the need for a reference-guided assembly analysis in order to create a high-quality genome assembly of Fv10027 (Table 5). To address this challenge, we developed a specific in-house bioinformatics tool: Reconstructor. This tool provided an automatic in silico approach aiming at generating a full genome sequence of an individual starting from the reference genome and resequencing data. This pipeline is based on two main steps: iterative read mapping and the de novo assembly as described in the Methods section (Figure 7). This step resulted in being crucial for unequivocally evaluating the variations in the genome of transformed strains compared to our wild type strain Fv10027. In fact, using as a reference the genome of Fv10027, we were able to eliminate the background noise in the variant calling analyses and focus our attention on the differences among Fv10027 and our transformant strains. The Fv10027_t1 genome served to generate a reference-guided assembly (Fv10027_t1), with which it was possible to create a reference genome of our strain to enhance the precision of downstream analysis and to create a stable reference for further genetic study.

## 3. Discussion

Fungi are organisms with an incredible plasticity that allow themselves to adapt to the most variable environmental conditions. Fungi may switch shapes, metabolism and reproductive strategies accordingly to several external stimuli, such as the carbon substrates, colony age, temperature, light cycle, substrate type and/or in the presence of a host [26,27]. The pleomorphic characteristic of fungi [28] is also known as “phenotypic plasticity”, that is the ability of any organism to respond to environmental signals by altering morphology, physiological state or behavior [29]. In phytopathogenic fungi, the phenotypic plasticity closely relates to the genotypic versatility, since the genome itself may adapt to environmental clues (epigenetic memory; [27]). However, relatively little is known about the genetic basis of plasticity, even if genes with specific functions (e.g., related to defense) are more “plastic” than others (i.e., DNA repair; [30]). 

*F. verticillioides*, a fungal pathogen of important crops such as maize, seldom reproduces sexually in nature, while often producing a huge amount of asexual spores during its infection or endophytic cycle in the host [31]. In such fungi, variability may be achieved through the parasexual cycle. As specified in the Introduction, these cycles occur in compartments (septate hyphae) where for several reasons (e.g., hyphal anastomosis), multiple nuclei are present. These nuclei may fuse and give rise to, for instance, aneuploid variants [6]. Moreover, specific conditions, such those experienced under in vitro cultures, produce a certain degree of genetic variability, probably by mitotic recombination [8]. 

In this study, we suggest that these natural processes of generating genetic variations without sexual reproduction are present in *F. verticillioides*, as well. In fact, subculturing procedures enhance the variants’ accumulation and trigger nuclear heterozygosity (e.g., multiple haplotypes). We found that the “standard” variations, the rate of in vitro propagated *F. verticillioides* (Fv10027) is ~14 variants/subculture. This means of genetic variations is enhanced considerably in mutant strains generated by deleting the *lds1* gene for homologous replacement with a selectable cassette inserted by PEG-transforming Fv10027_t1 protoplasts. Namely, Figure 4 clearly indicates that in specific, highly significant, loci, the transformation (independently of the construct inserted) “weight” is approximately 500 up to 1800 variations per single event (according to the nature of cassette insertion, i.e., through homologous or non-homologous recombination as in FvHph^+^). Our idea is that the procedures normally accomplished for deleting a gene destabilize the entire genome by enhancing means of naturally occurring variations in *F. verticillioides* as indicated by variations in the genotype and phenotype of FvHph^+^. The different phenotypes arisen from the single deletion of *lds1* may be thus ascribed to the enhancement of these variability-driving forces by the transformation event. Notably, even if a close relation between gene function and phenotypes cannot be stated at this stage, we noticed that the expression of the genes affected (e.g., FVEG_07317 and FVEG_07318 involved in energy utilization) by some variants in the *lds1*-deleted strains is significantly altered. As stated by other authors, “phenotypic plasticity is associated with a gene’s biological function and those genes involved in energy utilization, protein production and defense had high phenotypic plasticity” [30].

Nevertheless, the main focus of the study is to elucidate the bases of genome perturbation; with this research, a major breakthrough has been made in the field of fungal bioinformatics. For the first time, we used an integrated approach to create a private genome version allowing us to have many advantages. The main advantages we have exploited are: an increasing efficiency in the downstream analysis as marker development, ploidy studies and phylogenetic analyses, a better design of molecular experiment (primer design, PCR and RT-PCR), a better efficiency in the bioinformatics analysis (mapping rate increase and better variant calling) and the creation of a specific genomic resource for further studies. In the genomics, this is not the first example of the use of a combined strategy. Indeed, Gan et al. in 2011 [32] already developed a similar tool for plants and animals called IMR/DENOM. Reconstructor has been developed using this basic idea, but adding several innovative features that make it unique: an improved mapping efficiency using the bwa-mem algorithms, an improved variant caller system specifically developed to fine call heterozygous variations and an improved de novo insertion system that allows us to create a low error private genome.

In conclusion, the main finding of this study would stimulate a certain caution in considering the implication of gene study-of-function carried out by the gene knock-out strategy. There emerges a scenario in which at least PEG-mediated transformation and recombination (homologous or non-homologous) with a deletion/selectable marker cassette randomly destabilize the entire genome even affecting the expression of genes involved in primary metabolism; hence the need to have a specific physiological marker regarding the expected effect of gene deletion and then to consider the features of the phenotype not fully ascribable to the gene deletion. Further studies are needed for elucidating which is the main actor of genome perturbation: the trivial transformation event, the presence of a selective agent (hygromycin B), the disruption cassette and if other types of transformation (i.e., *Agrobacterium*-mediated) may be “kinder” to the host genome.

## 4. Experimental Section

### 4.1. Fungal Strains and Media

*F. verticillioides* wild type strain, deposited in the fungal germplasm bank of ISPA-CNR (Bari, Italy) with the Accession Number 10,027, was grown as monoconidial spore in potato dextrose agar (PDA) plates. The wild type strain was named Fv10027_t0, in order to indicate the starting point of subculturing studies. The Fv10027_t0 strain was subcultured twenty times, occurring every fifteen days, named Fv10027_t1, and used for *lds1* deletion. The Fv10027_t1 was transformed by homologous recombination, and fifty *lds1*-deleted (Δ*lds1*) strains were obtained. They were grouped according to colony morphology into four groups. One representative from each group was chosen for the in vitro analysis (ΔFv_lds1D, L, M and T). Based on their physiological and morphological features, two Δlds1 strains named ΔFv_*lds1*D and ΔFv_lds1T, deleted in a single copy of the lds1 gene [16], were further selected for the in vivo analysis and for the NGS analysis. For in vivo analysis, fungi were inoculated on maize ears as described in Scala et al. [16], whereas for nucleic acid extraction, fungi were also grown in 50 mL of liquid medium [33,34]. Fv10027_t1 was used to generate the FvHph^+^ control mutant. The transformation with the pAN7.1 vector (6.7 Kb), containing the cassette with the marker, was performed as described in Scala et al. [16]. The selection and purification of putative FvHph^+^ was performed by transferring the Hph resistant colonies onto PDA plates amended with 250 g/mL hygromycin B (Roche, Basel, Switzerland). Cassette integration was verified by Southern blot analysis (Supplementary Figure S3). The Southern blot was performed as reported in Scala et al., 2014 [16].

### 4.2. Physiological Analyses

The following parameters were evaluated in each strain: antioxidant enzymes, oxylipins (compounds and related gene expression), fumonisins amount, growth, conidiogenesis and conidia germination rate. Mean values are reported as variations in the parameters observed and calculated as the fold change of the *lds1*-deleted strains (ΔFv_*lds1*D and ΔFv_*lds1*T) over the WT, Fv10027_t1 strain. Antioxidant enzymes are calculated as the bulk of activities of superoxide dismutases, catalases and glutathione peroxidases expressed by the single strains of from 2 up to 15 days after inoculation (dai). Oxylipin-related genes expression (*lds1* excluded), after 5, 7, 10 and 15 dai and, namely, *lds2*, *lds3* and *lox* (here, gathered as the sum), were analyzed in RT-qPCR and 2^−ΔΔCt^ values calculated using FV10027 as the calibrator, with β-tub as the housekeeping gene. Several LDS-derived and LOX-derived oxylipins (considered as the sum of their respective amount) were quantified by LC-MS/MS. The growth of *lds1* mutants under in vitro conditions was calculated by radial diameter evaluation from 0 to 7 dai, whereas the growth in maize ear was calculated by qPCR. Conidiogenesis (conidia/mL) was evaluated at 15 dai and conidia germination (%) at 24 h after inoculation (hai). FB production was analyzed by LC-MS/MS at 15 dai. Some of these parameters and, namely, fungal growth, conidiogenesis, oxylipin gene expression and fumonisins biosynthesis were monitored in FvHph^+^, as well All of the methods are described in Scala et al. [16], Ludovici et al. [35] and Reverberi et al. [27]. A complete list of data per strain (Fv10027 included) is reported in Supplementary Table S1.

### 4.3. Sequencing

For each strain, ΔFv_*lds1*D, ΔFv_*lds1*T, FvHph^+^, Fv10027_t0 and Fv10027_t1, 100-bp reads paired-end NextEra libraries, with an average insert size of 500 bp, were produced and sequenced with the Illumina HiSeq 1500 technology at GenomiX4life (Baronissi, Italy). 

### 4.4. Bioinformatic Analysis

#### 4.4.1. Filtering of Sequencing Data

The raw FASTQ Illumina data were processed to understand and evaluate the reads using FastQC v0.11.2. FastQC was used before and after the filtering process to evaluate the quality of the raw reads. The filter and trimming processes were made with Trimmomatic v0.33 with leading: 25, trailing: 25, head crop: 13, sliding window: 28 and minimum length: 35 filtering option. In the FASTQ Illumina raw data files of all of the samples, an average of 7.19 × 10^6^ raw reads were obtained with a read length of 100 bp (150 bp for FvHph sample) and with a GC content of 48%. The trimming stage was used to crop Illumina raw data, as well as to remove adapters and to filter and trim the low quality reads present. After this stage, on average, 23.6% of the entire read set was discarded, and the mean read length decreased at 90 bp (140 bp for FvHph^+^ sample) with a minimum reads length of 35 bp.

#### 4.4.2. SNP, DIP and SV Calling

Variant calling has been performed by SUPER-W v4 available at superw.sequentiabiotech.com. SUPER-W is an open-source, dynamic and fast tool to analyze the variation data produced from the resequencing experiments [24]. SUPER uses Samtools (v1.2) to call small variations (such as SNPs and DIPs) and the lumpy tool (v.0.2.11) for the SVs (including deletions, inversions, duplications and translocations). SNPs and DIPs filtering was first performed using SnpSift v3.6c. This tool set works on the Variant Calling File (vcf) format. The applied filter included a minimum depth of 5 reads, PHRED quality of 30 and a homozygous value 0.8 (quality of variation, QUAL > 30; coverage of variation, DP ≥ 5; allele frequency, AF1 ≥ 0.8). The vcf file data were analyzed using vcf-compare (VCFtools v0.1.12b) and R v3.1.1 tools to highlight common and individual variations among Fv10027_t0, ΔFv_*lds1*D, ΔFv_*lds1*T and FvHph^+^ strains. A manual filter, using bash scripting, was applied to decrease the number of false positives found. The SVs was called with SUPER that uses the lumpy v0.2.11 tool. The lumpy result file was filtered to extract unique SVs for each strain analyzed. Variations were analyzed by SnpEff v3.6c, a tool to annotate the effect of each variant and create global statistics about variation calling. The results were visualized by the user using IGV v2.3.34. The single variations were individually analyzed visualizing the change-related coordinates of every snapshot produced by IGV.

#### 4.4.3. Genome Reconstruction

The reconstruction analysis has been performed using *F. verticillioides* 7600 (Fusarium Research Center, FRC M3125 = ARS Culture Collection, NRRL 20956) as the reference genome released by the Broad Institute and the filtered sequencing data of our wild strain Fv10027_t0. *F. verticillioides* 7600 is estimated to be 41.7 Mb with 12 chromosomes with 14,179 coding genes [23]. We have used the Fv 7600 reference genome with scaffolds: 36 scaffolds, 41.7 Mb and 15,869 genes. The genome reconstruction analysis was carried out using the Reconstructor tool (v.1.0). The Reconstructor pipeline is based on two main steps: iterative read mapping and de novo assembly. The first one is based on SUPER-W [24], a dynamic and fast tool to identify sequence variations, such as SNPs, DIPs and structural variations (SVs). Using a recursive approach, SNPs and SVs are added to a reference genome. The second step uses a de novo assembly strategy combined with BLAST search (local alignment routine) for the generation of species-specific contigs. Using paired-read and split read approaches, Reconstructor integrates the sequence variants identified in the first step with the new contigs produced in the de novo assembly in order to obtain a new genome sequence. Starting from the complete reconstruction of the genome of Fv10027, the gene annotations were transferred from the reference genome (Fv7600) to the new one by using PASA v2.0.2. Lastly, assembly statistics were calculated for the reconstructed genome sequences in order to assess the overall quality of the assembly (Table 5). A small amount of variants due to their low quality have not been added to the reconstructed genome.

During the iterative read-mapping step, the following filters were applied to the detected variants before their integration to the genome sequence: QUAL > 30, DP ≥ 6, AF1 = 1.0. On the other hand, in the de novo assembly step, only those contigs larger than 400 bp, with a fungi BLAST hit and with a minimum junction support of 4 read pairs per corner of the contig, were inserted into the reconstructed genome. For the genome annotation, we used PASA, a eukaryotic genome annotation tool that exploits spliced alignments of expressed transcript sequences to maintain gene structure annotation consistent between genome assemblies automatically. Only those transcripts/genes with a minimum alignment of the 90% with a minimum identity of 95 were transferred. 

### 4.5. Molecular Analysis

#### 4.5.1. Nucleic Acid Extraction and cDNA Synthesis

For each strain, genomic DNA was extracted as described in Scala et al. [16] and RNA by using the Plant/Fungi RNA Purification kit according to the manufacturer (Norgenbiotek, Thorold, ON, Canada). DNA and RNA were quantified using Qubit^®^ 2.0 fluorimeter (Thermofisher Scientific, Waltham, MA, USA) and using respectively the Qubit^®^ dsDNA BR Assay kit and Qubit^®^ RNA Assay kit (Thermofisher Scientific, Waltham, MA, USA). Complementary DNA (cDNA) was obtained using the Tetro cDNA Synthesis kit using an Eppendorf Mastercycler^®^ gradient (Eppendorf, Hamburg, Germany).

#### 4.5.2. Transcript Quantification Assay

Total RNA, from the mycelia of Fv10027_t1, ΔFv_*lds1*D, ΔFv_*lds1*T, L and M strains, was extracted at 2 to 7 dai and used to perform a reverse transcription PCR (RT-PCR). cDNA was used as a template for the qPCR experiments of different genes, as reported in Table 5. SYBR green (SensiMixTM SYBER No-ROX Kit (BIOLINE, Trento, Italy) qPCR amplification was performed in a Line Gene 9620 thermocycler (Bioer, Hangzhou, Zhejiang Province, China) as specified elsewhere [16]. Gene expression in the Fv10027_t1, ΔFv_*lds1*D, ΔFv_*lds1*T, L and M strains was calculated by using the 2^−ΔΔCt^, by normalizing the transcript levels of the gene of interest to the transcript of a reference gene (β-Tubulin, FVEG_ 04081.5) and calibrating the relative expression of the same gene in Fv10027_t1 [36]. 

#### 4.5.3. Morphological Analyses by Fluorescence Microscopy

For microscopic analysis, Fv10027_t0 wild type strain was grown for 24 h on coverslips with yeast extract glucose (YEG) medium and transferred to 3.7% formaldehyde, 50 mM phosphate buffer (pH 7) and Triton X-100 for fixation and incubated at room temperature for 30 min. After a rinse in distilled water, the coverslip was incubate for 5 min in a solution containing calcofluor and DAPI for staining. The mycelium was observed using a fluorescence microscope (Zeiss, Oberkochen, Germany) with a 100× objective. The digital image was acquired with a digital camera and elaborated using GNU Image Manipulation Program (GIMP) v.2.8.14. 

### 4.6. Statistical Analysis

All statistical analysis was performed by R v3.1.1 and Rstudio v0.99.467 statistical software tools and XLSTAT Version 2015.3.01.19199 (Addinsoft, Paris, France) [37]. In each qPCR experiment, datasets were pooled and compared using Student’s *t*-test, and the differences were considered significant when the *p*-value was <0.05.

## Figures and Tables

**Table 1:** Variations of some physiological parameters in *lds1*-deleted and control mutant (FvHph^+^) strains of *F. verticillioides* compared to Fv10027 under in vitro (liquid medium) and in vivo (maize ears), conditions (check the experimental section).

Strains	Antioxidant Enzymes	Oxylipin Genes	Oxylipins	Growth	Conidia	Conidia Germination	FBs
**In vitro**							
ΔFv_*lds1*D	0.20 ± 0.02	29.9 ± 1.2	0.52 ± 0.02	0.95 ± 0.11	0.74 ± 0.08	0.72 ± 0.05	3.4 ± 0.2
ΔFv_*lds1*T	0.24 ± 0.04	63.5 ± 5.3	0.54 ± 0.05	0.88 ± 0.05	2.22 ± 0.15	0.99 ± 0.08	3.2 ± 0.2
ΔFv_*lds1*M	0.31 ± 0.02	2.9 ± 0.5	0.62 ± 0.04	0.97 ± 0.02	0.60 ± 0.04	1.21 ± 0.12	1.0 ± 0.1
ΔFv_*lds1*L	0.19 ± 0.04	30.7 ± 5.2	0.49 ± 0.03	0.92 ± 0.08	1.07 ± 0.14	1.21 ± 0.09	3.2 ± 0.2
FvHph^+^	--	17.3 ± 4.2	--	0.68 ± 0.10	1.01 ± 0.07	--	1.3 ± 0.1
**In vivo**							
ΔFv_*lds1*D		9.9 ± 1.5	1.1 ± 0.2	0.76 ± 0.04			15.2 ± 3.2
ΔFv_*lds1*T		57.5 ± 5.1	1.3 ± 0.2	1.56 ± 0.22			139.4 ± 16.3

**Table 2:** Variant calling analysis using the reconstructed Fv10027_t1 genome as a reference highlights genetic variations with the wild type (Fv10027_t0) and three mutant strains (ΔFv_*lds1*D and ΔFv_*lds1*T, FvHph^+^). DIP, deletion/insertion polymorphism.

ALL Variants	Raw Variant	Quality Filtered and Background Removed	Referenced to Fv_10027_t1
Total variants	285,588	18,964	13,202
Total SNPs	273,744	18,802	13,086
Total DIPs	11,844	162	116

**Table 3:** Mutant strains’ high quality variation. For each variation, the position in the reconstructed genome, the gene or the genes affected and the potentially effects on it are presented. Variations are classified by type: SNPs (single nucleotide polymorphism), DIPs (deletion/insertion polymorphism) and SV (deletions, inversions and duplications, structural variation).

Name of Variant	Position	Gene Name	Type of Variant	Description of Gene	Predicted Function
SNP1	Scaffold_2:1748174	FVEG_14979	stop_gained	hypothetical protein	
SNP2	Scaffold_3:383277	FVEG_02691	stop_gained	amidohydrolase ytcJ-like	
SNP3	Scaffold_3:985831	FVEG_02859	stop_gained	hypothetical protein	
SNP4	Scaffold_4:2239365	FVEG_04184	stop_gained	hypothetical protein	GPI-anchor transamidase
SNP5	Scaffold_5:2242191	FVEG_05070	stop_gained	hypothetical protein	THUMP domain, involved in RNA metabolism
SNP6	Scaffold_9:1378671	FVEG_07628	stop_gained	hypothetical protein	
SNP7	Scaffold_12:878190	FVEG_09192	stop_gained	hypothetical protein	Myc-type, basic helix-loop-helix DNA binding protein
SNP8	Scaffold_13:426900	FVEG_09582	stop_gained	hypothetical protein	
SNP9	Scaffold_13:1337782	FVEG_09864	stop_gained	hypothetical protein	AMP-dependent synthetase/ligase; phosphopantetheine binding ACP domain
SNP10	Scaffold_15:1311389	FVEG_10951	stop_gained	hypothetical protein	Proton-dependent oligopeptide transporter family
SNP11	Scaffold_17:682448	FVEG_11590	stop_gained	iron-sulfur clusters transporter ATM1	
SNP12	Scaffold_18:160090	FVEG_11865	stop_gained	hypothetical protein	Zn(2)-C6 fungal-type DNA-binding domain; transcription factor domain, fungi
SNP13	Scaffold_19:97648	FVEG_12210	stop_gained	hypothetical protein	
SNP14	Scaffold_19:261693	FVEG_12278	stop_gained	hypothetical protein	
SNP15	Scaffold_19:418919	FVEG_17230	stop_gained	hypothetical protein	
DIP1	Scaffold_4:1048999	FVEG_03822	frameshift	hypothetical protein	Basic region leucine zipper
DIP2	Scaffold_1:2640553	FVEG_00886	frameshift	hypothetical protein	Leucine-rich repeat domain
DIP3	Scaffold_4:2461229	FVEG_04252	frameshift	hypothetical protein	Metallo-beta-lactamase
DIP4	Scaffold_22:131177	FVEG_13121	Upstream gene variant	hypothetical protein	Sensitivity to red light reduced-like, SRR1
	Scaffold_22:131177	FVEG_13122	Disruptive in-frame deletion	hypothetical protein	
	Scaffold_22:131177	FVEG_13123	Downstream gene variant	hypothetical protein	WD40-repeat-containing domain
DIP5	Scaffold_5:1387592	FVEG_04792	frameshift	hypothetical protein	Ribosome biogenesis protein Nop16
DIP6	Scaffold_9:391617	FVEG_07297	frameshift	hypothetical protein	MIF4G-like, type 3 domain; armadillo-type fold domain
DIP7	Scaffold_12:1355894	FVEG_09359	frameshift	hypothetical protein	transmembrane transport
DIP8	Scaffold_17:18487	FVEG_11383	frameshift	calcium binding protein 39	
DIP9	Scaffold_17:18487	FVEG_11384	frameshift	hypothetical protein	SWIB domain
DIP10	Scaffold_18:298218	FVEG_11924	frameshift	hypothetical protein	
DIP11	Scaffold_23:269982	FVEG_13429	frameshift	hypothetical protein	S-adenosyl-l-methionine-dependent methyltransferase
Structural Variants present only in ΔFvl_ds1T and ΔFv_lds1D stains					
SV1	Scaffold_9:449257	FVEG_07317	stop lost, in-frame deletion, splice region variant	adenosine triphosphatase	ATPase family associated with various cellular activities
	Scaffold_9:449257	FVEG_07318	Upstream gene variant	cystathionine beta-lyase	Cys/Met metabolism, pyridoxal phosphate-dependent enzyme

**Table 4:** Number of variants and haplotypes (e.g., zygosity) in Fv10027_t0, ΔFv_*lds1*D and ΔFv_*lds1*T with respect to Fv10027_t1. The “total variants” row refers to the total number of variant sites identified across the analyzed strains.

ALL Variants	Fv10027_t0	Fv_Hph^+^	ΔFv_*lds1*D	ΔFv_*lds1*T
Total variants	289	1833	542	1409
Heterozygous variants	289	1825	541	1408
Homozygous variants	0	8	1	1

**Table 5:** Assembly statistics of the Fv10027 genome reconstruction.

Number of Scaffolds	36
Total size	41,749,604 bp
Longest sequence	4,625,635 bp
Shortest sequence	14,062 bp
Mean sequence size	1,159,711 bp
N50	1,960,296 bp
Number of genes	15,867
Number of transcripts	20,551

## Supplementary Materials

The following are available online at www.mdpi.com/2072-6651/9/6/183/s1. Figure S1. Characterization of the genomic organization of Hph cassette in ΔFv_lds1D (10 µg genomic DNA; (A) and (5 µg genomic DNA; (B), and ΔFv_lds1T (10 µg genomic DNA; (C) and (5 µg genomic DNA; (D). Southern Blot hybridization of KpnI-restricted genomic DNA was carried out using PCR digoxigenin (DIG)-labelled fragments by using primers Hph_pAN7.1_For (5′-AACTGTGATGGACGACACCG) and Hph_pAN7.1_Rev (5′-GATTTGTGTACGCCCGACAG). Hph probe was hybridized at 50 °C. Molecular weight DNA marker: DIG-labeled λhindIII. Figure S2. Validation of NGS information by standard procedures. (A) Sanger sequencing of the DIP1 variant; (B) Sanger sequencing of the DIP4 variant; (C) Sanger sequencing of the SNP5 variant; SV-1 validation by end-point PCR (Lane 1 Fv10027_t1, Lane 2 ΔFv_lds1D and Lane 3 ∆Fv_lds1T); (D) The Integrative Genomics Viewer (IGV) of the entire samples set (from the first row: Fv10027_t0, Fv10027_t1, FvHph^+^ ∆Fv_lds1D and ∆Fv_lds1T) showing the 535 bp SV, in the position “Scaffold_9:449256”. Figure S3. Characterization of the genomic organization of the *Hph* cassette in FvHph^+^ (A,B), Fv10027_t1 (C,D), positive control (pAN 7.1::*Hph*) (E). Southern Blot hybridization of *Kpn*I-restricted genomic DNA was carried out using PCR digoxigenin (DIG)-labelled fragments by using primers Hph_pAN7.1_For (5′-AACTGTGATGGACGACACCG) and Hph_pAN7.1_Rev (5′-GATTTGTGTACGCCCGACAG). The *Hph* probe was hybridized at 50 °C. Table S1: Dataset concerning the physiological parameters of Fv10027, ΔFv_*lds1*D, ΔFv_*lds1*T, L and M. These values were used for performing PCA deriving from the dataset exemplified in Table 1; we use a Pearson correlation coefficient. Thus, the distance among centroids representing the single strains provides the significance of difference among the Eigen values of the PCs [37]. Table S2: List of primers used for cDNA amplification. Table S3: Number of reads of the samples (Fv10027_t0, Fv10027_t1, FvHph^+^, ΔFv_*lds1*D and ΔFv_*lds1*T) obtained on the Illumina HiSeq platform, before and after the trimming.
