# Supplementary material for: Careful with That Axe, Gene, Genome Perturbation after a PEG-Mediated Protoplast Transformation in Fusarium verticillioides
Source: Toxins (Basel). 2017 May 31;9(6):183. doi: 10.3390/toxins9060183 (PMC5488033; doi:10.3390/toxins9060183)
Supplement: Supplementary file 1 [file toxins-09-00183-s001.pdf]

Supplementary Materials: Careful with That Axe, Gene. Genome Perturbation after a PEG-Mediated Protoplasts Transformation in *Fusarium verticillioides*

Valeria Scala, Alessandro Grottoli, Riccardo Aiese Cigliano, Irantzu Anzar, Marzia Beccaccioli, Corrado Fanelli, Chiara Dall'Asta, Paola Battilani, Massimo Reverberi and Walter Sanseverino

Table S1. Phenological and physiological characterization of FvHph+ strain in comparison with WT strain, Fv10027_t1.

strains	Dry Weight (mg)		Spores/mL	Fumonisins
	2 dai	5 dai	5 dai	5 dai
*Fv*10027_t1	153 ± 4	234 ± 9	6.73×10^6	47.2 ± 1.2
*Fv*Hph$^+$	118 ± 2	160 ± 0	6.81×10^6	60.3 ± 2.2

Table S2. List of primers used for cDNA amplification.

Gene		T. Annealing (°C)	Sequence
FVEG_13121	FW	65	TGGTTGATGCGAAGACCCTC
	REV		CTCCACGTTCTCGATGTGCT
FVEG_13122	FW	65	GGTCCCACCAACAATCCCTT
	REV		TTGTCGCCTGCCTTTACAGT
FVEG_13123	FW	66	GTAGTGACTGGTTGTGCCGA
	REV		ATTGTTCCGTCGTTGCTTGC
FVEG_07317	FW	67	CGTAGAAGTGGCGAGCATGA
	REV		AACCATGATTCGAGCAGGCA
FVEG_07318	FW	66	TCCCATGCTGTTCAACCCTC
	REV		AACACCAGCCATGATGTCGT
FVEG_03821	FW	67	CAGTAACCACGACGACCCAA
	REV		CGAGAAACTTCCCGAACGGA
FVEG_03822	FW	67	GGCTCCATCGTCATCTACCG
	REV		CAGCGTTCATCATCTTGGCG
β-TUB FW 65	FW	65	CTCTGCTCATTTCCAAGATCCG
	REV		GTAGTTGAGGTCACCGTAGGAGG

Table S3. Number of reads of the samples (Fv10027_t0, Fv10027_t1, FvHph+, ΔFv_lds1D and ΔFv_lds1T) obtained on the Illumina HiSeq platform, before and after the trimming.

	Before Trimming				
	Fv10027_t0	Fv10027_t1	FvHph$^+$	ΔFv_*lds1*D	ΔFv_*lds1*T
Reads	4718,572	3,473,791	9,385,919	7,028,131	11,346,389
Length	100	100	150	100	100
	After Trimming				
Reads	3,377,271	2,228,758	6,369,195	6,264,405	10,125,903
Length	35–90	35–90	35–140	35–90	35–90
Discard	28.43%	35.84%	32.14%	10.87%	10.76%

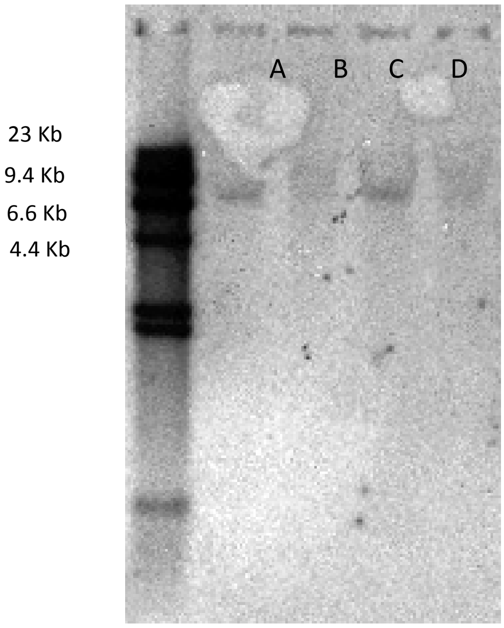

Figure S1. Characterization of the genomic organization of Hph cassette in ΔFv_lds1D (10 μg genomic DNA; (**A**) and (5 μg genomic DNA; (**B**), and ΔFv_lds1T (10 μg genomic DNA; (**C**) and (5 μg genomic DNA; (**D**). Southern Blot hybridization of KpnI-restricted genomic DNA was carried out using PCR digoxigenin (DIG)-labelled fragments by using primers Hph_pAN7.1_For (5′-AACTGTGATGGACGACACCG) and Hph_pAN7.1_Rev (5′-GATTTGTGTACGCCCGACAG). Hph probe was hybridized at 50 °C. Molecular weight DNA marker: DIG-labeled λhindIII.

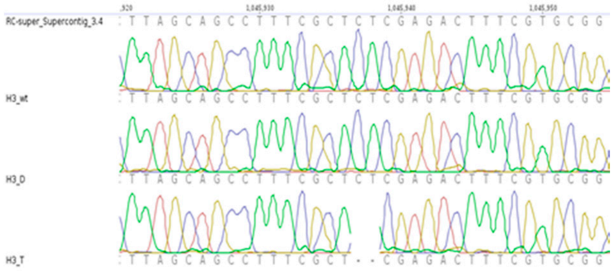

A

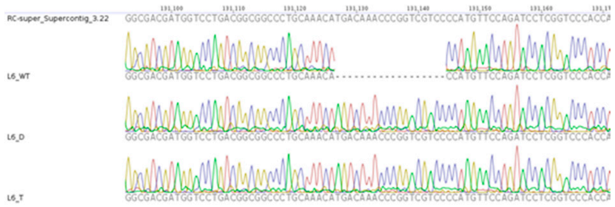

B

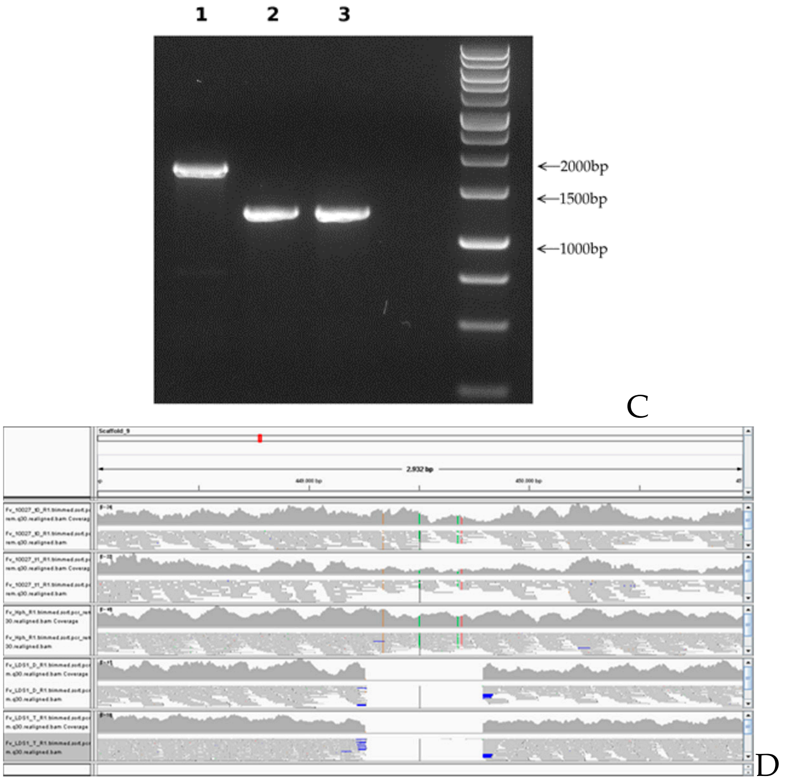

Figure S2. Validation of NGS information by standard procedures. A) Sanger sequencing of DIP1 variant; B) Sanger sequencing of DIP4 variant; C) Sanger sequencing of SNP5 variant; SV-1 validation by end-point PCR (lane1 Fv10027_t1, lane2 ΔFv_lds1D and lane 3 ΔFv_lds1T); D) The Integrative Genomics Viewer (IGV) of the entire samples set (from 1st row: Fv10027_t0, Fv10027_t1, FvHph+ ΔFv_lds1D and ΔFv_lds1T) showing the 535bp SV, in the position "Scaffold_9:449256".

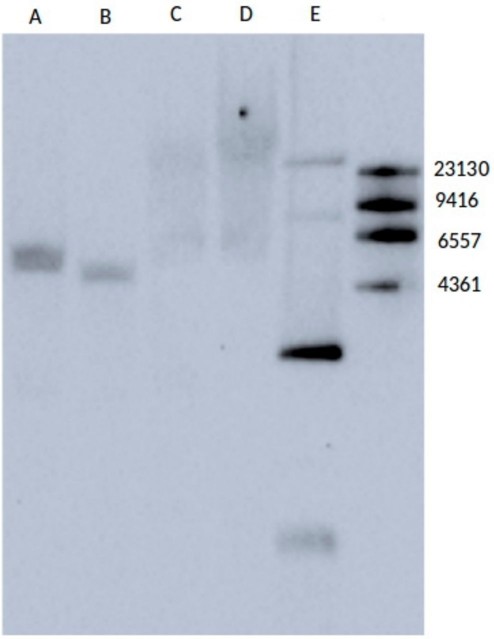

Figure S3. Characterization of the genomic organization of *Hph* cassette in FvHph+ (**A**,**B**), Fv10027_t1 (**C**,**D**), positive control (pAN 7.1::*Hph*) (**E**). Southern Blot hybridization of *Kpn*I-restricted genomic DNA was carried out using PCR digoxigenin (DIG)-labelled fragments by using primers Hph_pAN7.1_For (5′-AACTGTGATGGACGACACCG) and Hph_pAN7.1_Rev (5′-GATTTGTGTACGCCCGACAG). *Hph* probe was hybridized at 50 °C.
